# Safflorite, (Co,Ni,Fe)As_2_, isomorphous with marcasite

**DOI:** 10.1107/S1600536808026688

**Published:** 2008-08-23

**Authors:** Hexiong Yang, Robert T. Downs, Carla Eichler

**Affiliations:** a Department of Geosciences, University of Arizona, 1040 E. 4th Street, Tucson, AZ 85721-0077, USA

## Abstract

Safflorite, a naturally occurring cobalt-nickel-iron diarsenide (Co,Ni,Fe)As_2_, possesses the marcasite-type structure, with cations (*M* = Co + Ni + Fe) at site symmetry 2/*m* and As anions at *m*. The *M*As_6_ octa­hedra share two edges, forming chains parallel to *c*. The chemical formula for safflorite should be expressed as (Co,Ni,Fe)As_2_, rather than the end-member format CoAs_2_, as its structure stabilization requires the simultaneous inter­action of the electronic states of Co, Ni, and Fe with As_2_^2−^ dianions.

## Related literature

For related literature, see: Anawar *et al.* (2003 ▶); Carlon & Bleeker (1988 ▶); Darmon & Wintenberger (1966 ▶); Ennaciri *et al.* (1995 ▶); Goodenough (1967 ▶); Grorud (1997 ▶); Hem *et al.* (2001 ▶); Holmes (1947 ▶); King (2002 ▶); Kjekshus (1971 ▶); Kjekshus *et al.* (1974 ▶, 1979 ▶); Lutz *et al.* (1987 ▶); Makovicky (2006 ▶); O’Day (2006 ▶); Ondrus *et al.* (2001 ▶); Palenik *et al.* (2004 ▶); Petruk *et al.* (1971 ▶); Radcliffe & Berry (1968 ▶, 1971 ▶); Reich *et al.* (2005 ▶); Robinson *et al.* (1971 ▶); Swanson *et al.* (1966 ▶); Tossell (1984 ▶); Tossell *et al.* (1981 ▶); Vaughan & Rosso (2006 ▶); Wagner & Lorenz (2002 ▶).

## Experimental

### 

#### Crystal data

As_1.99_Co_0.61_Fe_0.17_Ni_0.22_S_0.01_
*M* *_r_* = 207.77
Orthorhombic, 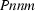

*a* = 5.0669 (6) Å
*b* = 5.8739 (7) Å
*c* = 3.1346 (4) Å
*V* = 93.29 (2) Å^3^
*Z* = 2
Mo *K*α radiation
μ = 43.75 mm^−1^
*T* = 293 (2) K
0.06 × 0.05 × 0.04 mm

#### Data collection

Bruker APEX2 CCD area-detector diffractometer
Absorption correction: multi-scan (*TWINABS*; Sheldrick, 2007 ▶) *T*_min_ = 0.179, *T*_max_ = 0.274 (expected range = 0.114–0.174)
1232 measured reflections
254 independent reflections
227 reflections with *I* > 2σ(*I*)
*R*_int_ = 0.041

#### Refinement

*R*[*F* ^2^ > 2σ(*F* ^2^)] = 0.025
*wR*(*F* ^2^) = 0.061
*S* = 0.91
254 reflections
13 parameters
Δρ_max_ = 1.44 e Å^−3^
Δρ_min_ = −1.82 e Å^−3^

### 

Data collection: *APEX2* (Bruker, 2003 ▶); cell refinement: *SAINT* (Bruker, 2005 ▶); data reduction: *SAINT*; program(s) used to solve structure: *SHELXS97* (Sheldrick, 2008 ▶); program(s) used to refine structure: *SHELXL97* (Sheldrick, 2008 ▶); molecular graphics: *XtalDraw* (Downs & Hall-Wallace, 2003 ▶); software used to prepare material for publication: *SHELXTL* (Sheldrick, 2008 ▶).

## Supplementary Material

Crystal structure: contains datablocks I, New_Global_Publ_Block. DOI: 10.1107/S1600536808026688/mg2054sup1.cif

Structure factors: contains datablocks I. DOI: 10.1107/S1600536808026688/mg2054Isup2.hkl

Additional supplementary materials:  crystallographic information; 3D view; checkCIF report

## Figures and Tables

**Fig. 1.**
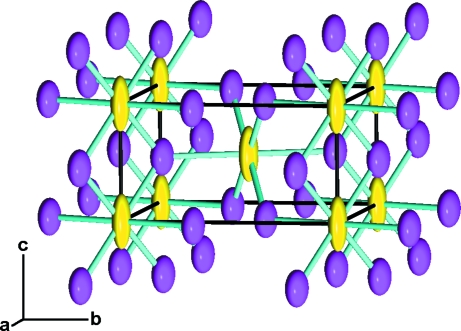
Crystal structure of safflorite, with displacement ellipsoids drawn at the 99.9% probabiliy level. The M (=Co+Ni+Fe) cations (yellow spheres) are situated in octahedra coordinated by six As atoms (pink spheres).

## supplementary crystallographic information

### Comment

Minerals in the FeAs_2_—NiAs_2_—CoAs_2 _system include löllingite FeAs_2_, rammelsbergite NiAs_2_, pararammelsbergite NiAs_2_, clinosafflorite CoAs_2_, and safflorite CoAs_2_. These diarsenide minerals, together with Fe—Ni—Co disulfides and sulfarsenides, are commonly found in complex Co—Ni—As ore deposits, such as Håkansboda, Sweden (Carlon & Bleeker, 1988), the Cobalt District, Ontario (Petruk *et al.*, 1971), Bou-Azzer, Morocco (Ennaciri *et al.*, 1995), Modum, Norway (Grorud, 1997), and Spessart, Germany (Wagner & Lorenz, 2002). When precipitated from hydrothermal solutions, these minerals can incorporate considerable amounts of trace metals, especially so-called ''invisible'' gold (*e.g.*, Palenik *et al.*, 2004; Reich *et al.*, 2005). Under oxidizing conditions, however, they can release significant amounts of arsenic into natural water and soils, in some cases producing serious arsenic poisoning and contamination (King, 2002; Anawar *et al.*, 2003; O'Day, 2006). Therefore, the crystal structures and bonding models of Fe—Ni—Co disulfides, diarsenides, and sulfarsenides have been a subject of extensive experimental and theoretical studies (Vaughan & Rosso, 2006; Makovicky, 2006, and references therein)

The crystal structures of all minerals, except safflorite, in the FeAs_2_—NiAs_2_—CoAs_2 _system have been determined. Topologically, löllingite FeAs_2_ (Kjekshus *et al.*, 1979; Lutz *et al.*, 1987; Ondrus *et al.*, 2001) and rammelsbergite NiAs_2_ (Kjekshus *et al.*, 1974, 1979) possess the marcasite (FeS_2_)-type structure with space group *Pnnm*, whereas clinosafflorite CoAs_2_ (Darmon & Wintenberger, 1966; Kjekshus, 1971) is isostructural with the modified marcasite-type structure of arsenopyrite (FeAsS) with space group *P*2_1_/*c* (Hem *et al.*, 2001; Makovicky, 2006). From the unit-cell dimensions measured from X-ray diffraction, safflorite was assumed to be isotypic with marcasite (Holmes, 1947; Radcliffe & Berry, 1968, 1971). Chemical analyses of various natural and synthetic samples reveal that *Pnnm* safflorite always contains some amounts of Fe and Ni, whereas materials with 80–100% (mole) CoAs_2_ crystallize in monoclinic *P*2_1_/*c* symmetry (Holmes, 1947; Swanson *et al.*, 1966; Radcliffe & Berry, 1971). This study presents the first structure determination of safflorite based on single-crystal X-ray diffraction data.

Safflorite is isomorphous with marcasite. Each cation (*M* = Co, Ni, and Fe) at site symmetry 2/*m* is octahedrally coordinated by six anions (As) at site symmetry *m* and each anion is tetrahedrally bonded to another anion (forming As—As dianion units) plus three *M* cations. The MAs_6_ octahedra share two edges, forming chains parallel to *c*, and two vertices with adjacent chains (Fig. 1). The average M—As bond distance (2.360 Å) is identical to that in clinosafflorite (Kjekshus, 1971), but slightly shorter than that in löllingite (2.379 Å) (Kjekshus *et al.*, 1979; Lutz *et al.*, 1987) or rammelsbergite (2.378 Å) (Kjekshus *et al.*, 1979). Notably, as the *d*-orbital electrons in *M* cations increase from Fe (*d* = 6) in löllingite to Co (*d* = 7) in safflorite, and Ni (*d* = 8) in rammelsbergite, the M—*M* separation along the chain direction increases significantly from 2.882 to 3.134, and 3.545 Å, respectively, while the As—As edge length shared by the two *M* octahedra concomitantly decreases from 3.808 to 3.547, and 3.219 Å. The octahedral distortion, measured by the octahedral angle variance (OAV) and quadratic elongation (OQE) (Robinson *et al.*, 1971), decreases. The OAV and OQE values are 92.87 and 1.0265 for FeAs_6_, 21.04 and 1.0058 for CoAs_6_, and 16.22 and 1.0049 for NiAs_6_.

The variation of the M—*M* separation with the number of *d*-orbital electrons in marcasite-type disulfides, diarsenides, and sulfarsenides has been a matter of discussion (see Vaughan & Rosso, 2006 for a thorough review). Theoretical calculations based on molecular orbital and band models predict that, due to the interaction between the 3*dσ*(*e_g_*) orbitals of *M*^2+^ and the *π_b_* orbitals of As_2_^2-^, the M—As—*M* angle subtending the M—*M* separation across the shared octahedral edge should be substantially smaller for FeAs_2_ than for CoAs_2_ and NiAs_2_, resulting in the so-called ''compressed marcasite-type'' structure (Tossell *et al.*, 1981; Tossell, 1984). Indeed, this angle is 74° in FeAs_2_ löllingite (Lutz *et al.*, 1987), but 83° in (Co,Ni,Fe)As_2 _safflorite and 96° in rammelsbergite (Kjekshus *et al.*, 1979). It is intriguing to note that the end-member CoAs_2_ has been found to only crystallize in the arsenopyrite-type structure (*P*2_1_/*c*) (Holmes, 1947; Swanson *et al.*, 1966; Radcliffe & Berry, 1971), rather than the marcasite-type structure (*Pnnm*). This observation may be explained by the existence of an unpaired electron occupying one of the *π_b_* orbitals, which splits into a lower-energy filled band and a higher-energy empty band (Goodenough, 1967), thus resulting in the symmetry reduction from *Pnnm* to *P*2_1_/*c*. In other words, the presence of some Ni/Fe in place of Co appears to be an essential requirement for the CoAs_2 _system to crystallize in the *Pnnm* symmetry. The pure system will otherwise be stabilized energetically in the clinosafflorite structure.

Another outstanding feature of the safflorite structure is the prominent anisotropic displacement ellipsoid of the *M* cation, the U_11_:U_22_:U_33_ ratio being approximately 3:1:9, with the ellipsoid axial directions roughly parallel to the unit cell axes. This ratio can be compared to the differences of three unit-cell dimensions between FeAs_2 _löllingite and NiAs_2_ rammelsbergite [(*a*_Lol _– *a*_Ram_)/*a*_Lol_: (*b*_Lol_ - *b_Ram_*)/*b*_Lol_: (*c*_Lol_ -*c*_Ram_)/*c*_Lol_] (Kjekshus *et al.*, 1974, 1979; Lutz *et al.*, 1987), which is about 3:1:8. Accordingly, the marked anisotropy of the displacement parameters of the *M* cation in safflorite is interpreted as a consequence of positional disorder with Fe and Ni occupying apparent different positions, which in turn results from the different interactions of their *d*-electrons with the As_2_^2-^ dianions.

### Experimental

The safflorite specimen used in this study is from Timiskaming County, Ontario, Canada, and is in the collection of the RRUFF project (deposition No. R070611; http://rruff.info), donated by James Shigley. The average chemical composition (15 point analyses), (Co_0.61_Ni_0.22_Fe_0.17_)_Σ__=1_(As_1.99_S_0.01_)_Σ__=2_, was determined with a CAMECA SX50 electron microprobe (*http://rruff.info*).

### Refinement

Due to the similar X-ray scattering powers for Co, Ni, and Fe, all cations were assumed to be Co and their site occupancies were not determined during the refinement. All crystals examined were twinned, with {011} as twin plane. The structure refinements were performed based on X-ray diffraction data collected from a twinned crystal, which were processed with *TWINABS * (Sheldrick, 2007). The ratio of two twin components is 0.73:0.27. The highest residual peak in the difference Fourier maps was located at (0.133, 0.370, 0.256), 0.85 Å from atom As, and the deepest hole at (0.133, 0.473, 0), 0.69 Å from As.

### Crystal data

**Table:**

As_1.99_Co_0.61_Fe_0.17_Ni_0.22_S_0.01_	*F*_000_ = 168
*M**_r_* = 207.77	*D*_x_ = 7.396 Mg m^−^^3^
Orthorhombic, *P**n**n**m*	Mo *K*α radiation λ = 0.71073 Å
Hall symbol: -P 2 2n	Cell parameters from 153 reflections
*a* = 5.0669 (6) Å	θ = 5.0–31.4º
*b* = 5.8739 (7) Å	µ = 43.75 mm^−^^1^
*c* = 3.1346 (4) Å	*T* = 293 (2) K
*V* = 93.29 (2) Å^3^	Granular, black
*Z* = 2	0.06 × 0.05 × 0.04 mm

### Data collection

**Table:**

Bruker APEX2 CCD area-detector diffractometer	254 independent reflections
Radiation source: fine-focus sealed tube	227 reflections with *I* > 2σ(*I*)
Monochromator: graphite	*R*_int_ = 0.041
*T* = 293(2) K	θ_max_ = 36.4º
φ and ω scan	θ_min_ = 5.3º
Absorption correction: multi-scan(TWINABS; Sheldrick, 2007)	*h* = −8→7
*T*_min_ = 0.179, *T*_max_ = 0.274	*k* = −8→9
1232 measured reflections	*l* = −5→5

### Refinement

**Table:**

Refinement on *F*^2^	Secondary atom site location: difference Fourier map
Least-squares matrix: full	*w* = 1/[σ^2^(*F*_o_^2^) + (0.0428*P*)^2^] where *P* = (*F*_o_^2^ + 2*F*_c_^2^)/3
*R*[*F*^2^ > 2σ(*F*^2^)] = 0.025	(Δ/σ)_max_ < 0.001
*wR*(*F*^2^) = 0.061	Δρ_max_ = 1.44 e Å^−^^3^
*S* = 0.91	Δρ_min_ = −1.82 e Å^−^^3^
254 reflections	Extinction correction: SHELXL, Fc^*^=kFc[1+0.001xFc^2^λ^3^/sin(2θ)]^-1/4^
13 parameters	Extinction coefficient: 0.016 (6)
Primary atom site location: structure-invariant direct methods	

### Special details

**Table:**

Geometry. All e.s.d.'s (except the e.s.d. in the dihedral angle between two l.s. planes) are estimated using the full covariance matrix. The cell e.s.d.'s are taken into account individually in the estimation of e.s.d.'s in distances, angles and torsion angles; correlations between e.s.d.'s in cell parameters are only used when they are defined by crystal symmetry. An approximate (isotropic) treatment of cell e.s.d.'s is used for estimating e.s.d.'s involving l.s. planes.
Refinement. Refinement of *F*^2^ against ALL reflections. The weighted *R*-factor *wR* and goodness of fit *S* are based on *F*^2^, conventional *R*-factors *R* are based on *F*, with *F* set to zero for negative *F*^2^. The threshold expression of *F*^2^ > σ(*F*^2^) is used only for calculating *R*-factors(gt) *etc*. and is not relevant to the choice of reflections for refinement. *R*-factors based on *F*^2^ are statistically about twice as large as those based on *F*, and *R*- factors based on ALL data will be even larger.

### Fractional atomic coordinates and isotropic or equivalent isotropic displacement parameters (Å^2^)

**Table:**

	*x*	*y*	*z*	*U*_iso_*/*U*_eq_	
M	0.0000	0.0000	0.0000	0.0130 (2)	
As	0.18637 (9)	0.36589 (7)	0.0000	0.01033 (17)	

### Atomic displacement parameters (Å^2^)

**Table:**

	*U*^11^	*U*^22^	*U*^33^	*U*^12^	*U*^13^	*U*^23^
M	0.0090 (4)	0.0033 (4)	0.0267 (4)	0.0001 (3)	0.000	0.000
As	0.0140 (3)	0.0046 (2)	0.0124 (2)	−0.00002 (14)	0.000	0.000

### Geometric parameters (Å, °)

**Table:**

M—As	2.3475 (5)	M—As^iii^	2.3669 (4)
M—As^i^	2.3475 (5)	M—As^iv^	2.3669 (4)
M—As^ii^	2.3669 (4)	M—As^v^	2.3669 (4)
			
As—M—As^ii^	88.016 (9)	M—As—M^vi^	125.085 (13)
As^i^—M—As^ii^	91.984 (9)	M^vi^—As—M^vii^	82.931 (17)
As^ii^—M—As^iv^	82.931 (17)	M—As—As^viii^	106.12 (3)
As^iii^—M—As^iv^	97.069 (17)	M^vi^—As—As^viii^	107.599 (2)

Symmetry codes: (i) −*x*, −*y*, −*z*; (ii) *x*−1/2, −*y*+1/2, *z*−1/2; (iii) −*x*+1/2, *y*−1/2, −*z*+1/2; (iv) *x*−1/2, −*y*+1/2, *z*+1/2; (v) −*x*+1/2, *y*−1/2, −*z*−1/2; (vi) −*x*+1/2, *y*+1/2, −*z*+1/2; (vii) −*x*+1/2, *y*+1/2, −*z*−1/2; (viii) −*x*, −*y*+1, −*z*.
